# 培美曲塞联合顺铂或卡铂治疗复发或转移性非小细胞肺癌63例疗效分析

**DOI:** 10.3779/j.issn.1009-3419.2011.01.11

**Published:** 2011-01-20

**Authors:** 伟 王, 立群 尚, 学昌 李, 军 李, 锋 文, 军强 刘

**Affiliations:** 100048 北京，中国人民解放军海军总医院胸外科 Department of Toracic Surgery, Naval General Hospital of PLA, Beijing 100048, China

**Keywords:** 肺肿瘤, 肿瘤转移, 复发, 培美曲塞, 顺铂/卡铂, Lung neoplasms, Neoplasm metastasis, Recurrence, Pemetrexed, Cisplatin/carboplatin

## Abstract

**背景与目的:**

对初治后复发或转移性非小细胞肺癌（non-small cell lung cancer, NSCLC）患者的治疗，需引入新的二线药物及治疗方法。培美曲塞为多靶点抗叶酸化疗药物，近来多用于NSCLC的治疗。本研究旨在探讨培美曲塞联合顺铂或卡铂方案治疗复发或转移性NSCLC的疗效以及不良反应。

**方法:**

经病理学或细胞学确诊的复发或转移性晚期NSCLC患者63例，其中男性40例，女性23例，中位年龄62岁。培美曲塞500 mg/m^2^第1天+顺铂30 mg/m^2^第1、2、3天或卡铂300 mg/m^2^第1天静脉滴注，每3周重复。评价疗效及不良反应。

**结果:**

63例中完全缓解1例，部分缓解5例，稳定36例，进展21例，疾病控制率为66.7%（42/63），中位生存期为9.0个月。中位无疾病进展时间为5.0个月，其中鳞癌为3.0个月，腺癌为5.5个月，非鳞癌优于鳞癌，差异有统计学意义（*P*=0.017）。主要不良反应为粒细胞下降、贫血和胃肠道反应。

**结论:**

培美曲塞联合顺铂或卡铂方案治疗复发或转移性NSCLC疗效确切，对非鳞癌更具有治疗优势。不良反应发生率低，耐受性较好。

## Abstract (en)

**Background and objective:**

Since the poor outcome for advanced lung cancer with frst-line chemotherapy, more efforts should be paid for treatment of advanced recurrent or metastasis non-small cell lung cancer (NSCLC) patients. Pemetrexed, as a multi-target antifolate chemotherapeutic drug, was approved for the second-line treatment of advanced NSCLC. The aim of this study is to evaluate the efcacy and side effects of pemetrexed combined with cisplatin/carboplatin in the treatment of advanced recurrent or metastasis NSCLC.

**Methods:**

Sixty-three advanced recurrent or metastasis NSCLC patients confirmed with pathology or cytology were enrolled in this study. Among them, 40 cases were male and 23 were female, with 62 years of median age. The combination regimen was patients received pemetrexed 500 mg/m^2^ on day 1 and cisplatin 30 mg/m^2^ on day 1, day 2 and day 3 or carboplatin 300 mg/m^2^ on day 1 by intravenous infusion, with 21 days as one cycle. All patients who received 2 or more cycles could be evaluated.

**Results:**

Only 1 case got complete response, with 5 cases partial response, 36 stable and 21 cases progressive. The overal control rate was 66.7% (42/63). The median survival time was 9.0 months, while the median progression-free survival was 5.0 months (3.0 months in squamous cell carcinoma; 5.5 months in adenocarcinoma). Tere was a signifcant difference between squamous cell carcinoma and adenocarcinoma (*P*=0.017). The common adverse effects were leucopenia, anemia and gastrointestinal response.

**Conclusion:**

Pemetrexed combined with cisplatin/carboplatin is effective and feasible for advanced recurrent or metastasis NSCLC.

肺癌是一种常见的肺部恶性肿瘤，其死亡率己占癌症死亡之首。其中65%-70%的患者确诊时为不宜手术的Ⅲb/Ⅳ期患者。而Ⅲb/Ⅳ期非小细胞肺癌（non-small cell lung cancer, NSCLC）治疗以化疗为主，标准的一线化疗方案是以铂类为主的联合方案（吉西他滨、紫杉醇、多西他赛、长春瑞宾）^[1]^，一线化疗能延长患者生存期，改善患者生存质量，但有效率仅30%-40%，中位生存期约10个月，因此对复发或初治无效患者的治疗，需引入新的药物及治疗方法。多靶点抗叶酸化疗药物培美曲塞于2004年2月被美国食品和药物管理局（Food and Drug Administration, FDA）批准用于恶性间皮瘤的治疗，2004年8月被批准用于晚期NSCLC的二线治疗。我院自2005年10月-2009年12月，应用培美曲塞治疗63例晚期复发性NSCLC，疗效确切，现报道如下。

## 1 对象和方法

### 1.1 研究对象

2005年10月-2009年12月我院收治的初治化疗失败或手术治疗后复发转移的NSCLC患者63例，均经病理学或细胞学证实，有可测量的临床观察指标，肝、肾功能及血象正常，近1个月内未接受其它抗肿瘤治疗。63例患者中男性40例，女性23例，年龄35岁-82岁，中位年龄62岁，其中腺癌41例，鳞癌18例，大细胞癌2例，鳞腺癌2例。肿瘤及转移部位：肺内原发灶、纵隔及浅表淋巴结、肺脏、肝脏、脑、骨、胸膜、恶性胸腔积液及皮肤。

### 1.2 既往治疗情况

Ⅲb/Ⅳ期NSCLC既往曾接受一线或二线化疗失败的患者45例，其中接受1个化疗方案治疗者34例；接受2个以上化疗方案患者11例；化疗方案主要是吉西他滨、紫杉醇、长春瑞滨、多西他赛与顺铂或卡铂等不同方案的联合治疗，其中曾接受靶向治疗者12例，曾行放射治疗者11例。

接受手术治疗后复发或转移的NSCLC 18例，术前分期Ⅱb期5例、Ⅲa期13例，术后均接受2个-4个周期化疗，化疗方案同上，6例曾行术后放疗。中位转移时间13个月，转移部位：纵隔淋巴结转移8例、肺内转移4例、脑转移3例、多发骨转移2例、胸膜转移1例。63例患者的临床资料见表 1。

**1:** 患者临床资料（*n*=63） Clinical characteristics of patients (*n*=63)

Characteristic	*n* (%)
Gender	
Male	40 (63.5)
Female	23 (36.5)
Age (years)	
Median	62
Range	35-82
Performance score	
0-1	58(92.1)
2	5 (7.9)
Histology	
Adenocarcinoma	41 (65.1)
Squamous cell carcinoma	18 (28.6)
Others	4 (6.3)
Previous therapy	
First or second-line chemotherapy	63 (100)
Previous operation	18 (28.6)
Previous target therapy	12 (19.0)
Previous radiotherapy	11 (17.5)

### 1.3 治疗方案

全部患者接受联合化疗。培美曲塞500 mg/m^2^第1天静脉滴注+顺铂30 mg/m^2^第1、2、3天静脉滴注；或培美曲塞500 mg/m^2^第1天静脉滴注+卡铂300 mg/m^2^第1天静脉滴注，每3周为1个周期。共完成198个周期，中位周期数为4个周期（范围1个-8个周期），其中3例患者联合用药4个周期后用培美曲塞单药维持1个-4个周期。用药前一周开始给予口服叶酸400 µg/d，持续到治疗结束；用药前1周给予维生素B_12_ 1, 000 µg肌内注射，每9周1次；用药前1天、当天口服地塞米松7.5 mg。

### 1.4 评定标准

疗效按照世界卫生组织（World Health Organization, WHO）实体瘤近期疗效评定标准，分为完全缓解（complete response, CR）、部分缓解（partial response, PR）、稳定（stable disease, SD）和进展（progressive disease, PD）；不良反应根据WHO关于抗癌药物毒性反应评定标准评价。

### 1.5 随访与统计学方法

对所有患者进行随访，随访主要采用门诊定期复查、电话询问等。总生存时间（overall survival, OS）从入院开始到患者死亡或最后一次随访日，无疾病进展期（progression-free survival, PFS）从用培美曲塞化疗开始至疾病进展或疾病尚未进展的末次随访时间。使用SPSS 16.0统计软件的*Kaplan-Meier*法统计生存期和无疾病进展期，并采用*Log-rank*检验组间差异，*P* < 0.05为差异有统计学意义。

## 2 结果

### 2.1 疗效

随访至2010年5月，随访时间3.4个月-26.8个月，平均随访时间18.1个月。63例中CR 1例，PR 5例，SD 36例，PD 21例，疾病控制率66.7%（42/63）。中位生存期为9.0个月（95%CI: 8.24-9.76），其中鳞癌8.5个月（95%CI: 7.94-9.06），腺癌10.0个月（95%CI: 8.65 - 11.36），鳞癌和腺癌相比差异无统计学意义（*P*=0.179）；1年生存率腺癌为21.7%（6/23），鳞癌为18.5%。中位无疾病进展时间5.0个月（95%CI：4.18-5.84），其中鳞癌3.0个月（95%CI: 1.56-4.45），腺癌5.5个月（95%CI: 4.32-6.68），鳞癌和腺癌相比差异有统计学意义（*P*=0.017）（图 1）。

**1:**
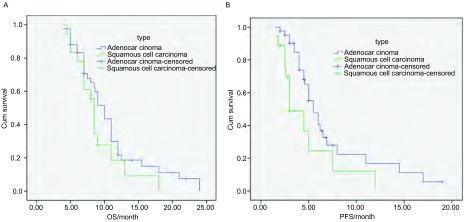
腺癌和鳞癌的生存期和中位无疾病进展时间比较。A：腺癌和鳞癌的生存期比较（*Log-rank*，*P*=0.179）；B：腺癌和鳞癌的中位无疾病进展时间比较（*Log-rank*，*P*=0.017）。 Comparison of overall survival (OS) and progression-free survival (PFS) between adenocarcinoma and squamous cell carcinoma of the lung. A: OS of adenocarcinoma and squamous cell carcinoma (*Log-rank*, *P*=0.179); B: PFS of adenocarcinoma and squamous cell carcinoma (*Log-rank*, *P*=0.017).

### 2.2 不良反应

63例患者均可评价毒性反应，Ⅰ/Ⅱ度和Ⅲ/Ⅳ度粒细胞减少发生率分别为53.9%（34/63）和12.7%（8/63），共使用粒细胞集落刺激因子（granulocyte colony stimulating factor, G-CSF）治疗10例（15.9%, 10/63），Ⅰ/Ⅱ度和Ⅲ/Ⅳ度贫血发生率为46.0%（29/63）和9.5%（6/63），Ⅰ/Ⅱ度血小板下降33.3%（21/63），Ⅰ/Ⅱ度和Ⅲ/Ⅳ度恶心、呕吐发生率分别为61.9%（39/63）和14.3%（9/63）。总体耐受性好，不良反应轻微。

## 3 讨论

培美曲塞是一个新的结构上含有核心为吡咯嘧啶基团的多靶点抗叶酸化疗药物，通过干扰细胞复制过程中叶酸代谢途径而发挥抗肿瘤作用。培美曲塞能够抑制几种与嘌呤和嘧啶分泌相关的叶酸依赖性酶，如胸苷酸合成酶（thymidylate synthase, TS）、二氢叶酸还原酶（dihydrofolate reductase, DHFR）和甘氨酰胺核苷甲酰基转移酶（dihydroflate reductase, GARFT）的活性，通过对这些关键酶活性进行多靶点抑制，使得嘌呤和胸腺嘧啶核苷生物合成减少，从而影响肿瘤细胞DNA和RNA合成^[2]^。

培美曲塞已表现出对多种肿瘤的抗癌活性，包括大肠癌、乳腺癌、肺癌、胰腺癌、间皮瘤及胃癌等^[3]^。尤其值得注意的是，培美曲塞对骨髓抑制的发生率和严重程度明显低于现在较为流行的一线化疗药物如多西他赛、吉西他滨、诺维本等。该药已被美国FDA批准为NSCLC的标准二线治疗方案^[4]^。随着培美曲塞广泛用于临床，由于其疗效较好、较低的累积毒性及较低的副反应得到了广泛的认可。鉴于此，FDA于2008年9月30日又正式批准增加培美曲塞的适应症，允许其与顺铂联用作为局部恶化和转移并伴有非鳞状组织学特性的NSCLC的一线治疗方案。而培美曲塞与铂类药物联合治疗晚期NSCLC的Ⅱ期临床研究^[5]^表明，培美曲塞/铂类联合方案的疗效与其它常用的含铂两药方案相似，而毒性反应的发生率则明显较低。Hanna等^[6]^进行的一项大型随机Ⅲ期临床研究显示，对比单药培美曲塞与多西紫杉醇二线治疗NSCLC，无论有效率（9.1% *vs* 8.8%）、中位生存期（8.3个月*vs* 7.9个月）还是1年生存率（均为29.7%），差异均无统计学意义，但是中性粒细胞下降、粒细胞性发热及脱发等药物不良反应在培美曲塞组明显降低。以上多项大型Ⅱ、Ⅲ期临床试验表明，培美曲塞方案疗效与其它一、二线方案相近，且不良反应低。鉴于此，本研究使用培美曲塞联合铂类治疗初治失败的或手术治疗后复发转移的NSCLC患者进行二线化疗，希望改善患者生活质量，延长患者生命。

本研究结果表明，63例中CR 1例，PR 5例，SD 36例，PD 21例，疾病控制率66.7%（42/63）。中位生存期为9.0个月，中位PFS为5.0个月，显示出培美曲塞联合铂类对初治失败或手术治疗后复发的NSCLC患者的较好疗效，与国外文献^[7, 8]^报道相似。

Scagliotti^[9]^于2008年报道了一项Ⅲ期随机对照临床研究，将1, 725例晚期NSCLC患者随机分为2组，分别接受顺铂+吉西他滨（*n*=863）或顺铂+培美曲塞（*n*=862）治疗，研究结果表明：在总生存期方面，顺铂+培美曲塞组不劣于顺铂+吉西他滨组。亚组分析表明：腺癌患者中，顺铂+培美曲塞组患者生存期优于顺铂+吉西他滨组患者（*n*=847；12.6个月*vs* 10.9个月），而在鳞癌患者中，顺铂+吉西他滨组患者生存期优于顺铂+培美曲塞组患者（*n*=473；10.8个月*vs* 9.4个月），该项研究首次表明培美曲塞对不同病理类型的肺癌其疗效具有差异。本研究中我们比较了鳞癌和腺癌的中位PFS，鳞癌3.0个月，腺癌5.5个月，鳞癌和腺癌相比差异有统计学意义（*P*=0.017），证实了培美曲塞对治疗非鳞癌有优势，与Scagliotti报道一致。中位生存期鳞癌8.5个月，腺癌10.0个月，虽然腺癌比鳞癌延长1.5个月，但两者无统计学差异，这有待于增大病例数并延长随访时间加以证实。

在毒副作用方面，Ⅲ/Ⅳ度血液学和非血液学毒副作用少见，可见粒细胞减少及消化道反应，但发生率分别为12.7%、14.3%，经升白细胞及对症治疗后均可恢复，因此在毒副作用方面显示出了良好的优势，所有患者并无因毒副作用不能耐受而停止化疗。即使体能状态评分为2的患者也显示出良好的耐受性和较佳的安全性。

总之，培美曲塞与顺铂或卡铂联合对初治失败或手术治疗后复发的晚期NSCLC患者可获得较高疗效，且毒性低、耐受性、安全性好，是目前拥有较为理想近期疗效的方案，值得在临床中加以应用。但该方案的远期疗效还有待进一步大规模随访、统计并加以总结。
